# Efficacy of Drug Interventions for Chemotherapy-Induced Chronic Peripheral Neurotoxicity: A Network Meta-analysis

**DOI:** 10.3389/fneur.2017.00223

**Published:** 2017-06-08

**Authors:** Xiying Fu, Huijie Wu, Jinyao Li, Can Wang, Ming Li, Qianqian Ma, Wei Yang

**Affiliations:** ^1^Department of Endocrinology, The Second Hospital of Jilin University, Changchun, Jilin, China; ^2^Department of Neurology, The Second Hospital of Jilin University, Changchun, Jilin, China

**Keywords:** chemotherapy-induced chronic neurotoxicity, network meta-analysis, efficacy, treatment, intervention

## Abstract

Peripheral neurotoxicity is a disturbing issue for cancer patients who are treated with chemotherapy. Several medications have been developed for preventing chemotherapy-induced chronic neurotoxicity (CICNT) however; their relative efficacies have not yet been studied. In this study, we conducted a network meta-analysis to give intervention recommendations. The literature was searched in a variety of databases and eligible studies were chosen based on predefined criteria. Data extraction and statistical analysis was performed, and the results are displayed using the odds ratio (OR) and corresponding 95% credible intervals (CrI) with respect to overall and severe neurotoxicity. The medications were ranked according to their surface under cumulative ranking curve values. The consistency of direct and indirect evidence was also evaluated. We found that patients with amifostine or vitamin E (VE) treatment exhibited a lower risk of overall neurotoxicity compared to those using the placebo (amifostine: OR = 0.10, 95% CrI: 0.02–0.46; VE: OR = 0.08, 95% CrI: 0.01–0.99). In regard to preventing severe neurotoxicity, glutathione and amifostine treatment appeared to be significantly more effective than the placebo (glutathione: OR = 0.19, 95% CrI: 0.04–0.64; amifostine: OR = 0.12, 95% CrI: 0.02–0.48). In summary, amifostine, VE, and glutathione treatment is considered to be effective in lowering the risk of CICNT. However, further studies which consider safety are required.

## Introduction

Chemotherapy is widely used as a cancer treatment; however, it induces peripheral neurotoxicity in patients (1). There are two main types of neuropathy that may be induced by different kinds of chemotherapies, acute neuropathy, and chronic neuropathy (2). Acute neuropathy normally only lasts 1 week, and it is not dose related to chemotherapeutics. Chronic neuropathy is dose-related and can lead to a more debilitating influence on patients by causing paresthesia or proprioceptive changes (3). As acute neuropathy is reversible and does not cause severe harm to the peripheral nervous system (2, 4–6), more attention is given to chronic peripheral neurotoxicity. In this study, we also focus on chronic neurotoxicity.

Various drugs are recommended to help prevent chemotherapy-induced chronic neurotoxicity (CICNT). Calcium and magnesium infusion, glutathione, amifostine, and vitamin E (VE) are the most favored treatments in terms of their effectiveness in weakening the neuropathy caused by cisplatin and other chemotherapeutics (7). The most widely used therapy for the prevention and treatment of CICNT is calcium and magnesium infusion. It is suggested to be implemented by approximately 50% of oncologists in their practices (8). However, the question of whether the use of calcium and magnesium infusion can actually reduce neurotoxicity remains controversial. Some studies (9, 10) proved its value in neuroprotection function while others (2, 8, 11) denied its effectiveness. Glutathione is also known as a promising and effective drug. Preliminary clinical trials which assessed the efficacy of glutathione demonstrated a decline in neurotoxicity and no negative interference in oncolytic activity (12, 13). Another organic thiophosphate drug called amifostine also displayed the capability to defend against the cytotoxic effects on tissues posed by chemotherapy and radiotherapy (14). It has also been proven to be neuroprotective when used in combination with various chemotherapies such as cisplatin (14), cyclophosphamide (14), oxaliplatin (15), and carboplatin (16). VE is an antioxidant that can eliminate free radicals in cells. Based on current studies, it is also believed to be able to protect against cisplatin-induced neuropathy (7, 17). A decreased VE level in plasma was detected in patients suffering from cisplatin-induced neuropathy (17). A recent study proposed that the supplementation of VE can significantly reduce peripheral nerve damage induced by cisplatin (neurotoxicity incidence rate of 21.4%, 68.5% in the controls) (18).

Despite the fact that the effectiveness of the above therapies has been confirmed by many trials, the lack of head-to-head comparisons is a main drawback of current literature. There is also paradoxical evidence regarding the efficacy and safety of these chemicals when applied to CICNT. This may partly result from different patient backgrounds, type of chemotherapy, or neurotoxicity scales. This inconsistency further adds to the complexity in correctly deducing a conclusion on their real effectiveness. Furthermore, the published meta-analyses mainly focus on the clinical comparisons between calcium and magnesium infusion, glutathione, and amifostine (7, 19–23). Therefore, they do not provide sufficient evidence on the relative efficacy of each drug. No intervention for CICNT has been generally accepted or identified based on MA outcomes (7, 24).

A network meta-analysis (NMA) also known as a multiple-treatment comparison allows for the synchronous extraction and analysis of data from medical trials. Unlike the conventional MA, it compares at least three interventions simultaneously and provides strong evidence on the relative efficacy of each treatment based on direct and indirect evidence (25, 26). This method has recently been utilized in many studies that aim to assess and compare the effectiveness of various therapeutic interventions (27–30). It also provides a useful and comprehensive summary that contributes to determining treatment.

By implementing an NMA in this study, our primary objective was to identify the most effective intervention to decrease the neurotoxicity caused by chemotherapy. We divided the related chronic neurotoxicity endpoints into two categories: the incidence of overall CICNT and the incidence of severe CICNT in patients. It should be noted that no NMA has been performed on the interventions of CICNT to date. Therefore, this study is quite necessary and may be very meaningful.

## Materials and Methods

### Literature Search

Published literature was first retrieved from the medical databases [Medline, Embase, and China National Knowledge Internet (CNKI)] regardless of language. The search terms were “neurotoxicity syndromes,” “calcium and magnesium,” “glutathione,” “vitamins,” “amifostine,” and “randomized controlled trial” and their synonyms. We removed duplicates, manually scanned the titles and abstracts, and reviewed the contents of studies to be involved. In order to prevent any omission, the reference lists were also examined. All the screening work was independently completed by two experienced researchers. If any disagreement arose, a discussion including a third party was performed to offer a mediated plan.

### Selection Criteria

There were four inclusion criteria: (1) the study contains the assessment of chemotherapy-induced neurotoxicity in cancer patients; (2) the study contains at least one pairwise comparison between the drugs (placebo, calcium and magnesium, glutathione, amifostine, and VE); (3) the study provides enough data, for example, the number of patients under each grade of neurotoxicity; (4) and the study assesses the incidence of chemotherapy-induced overall chronic peripheral neurotoxicity (referred to as overall neurotoxicity below) or the incidence of chemotherapy-induced severe chronic peripheral neurotoxicity (referred to as severe neurotoxicity below) as endpoints.

### Data Extraction

Two independent investigators were arranged to collect relevant data from each eligible study. Information consisting of name of author, publication year, type of randomization, type of blinding method, neurotoxicity assessment standard, tumor site, type of chemotherapy, type of intervention, sample size, dose as well as the overall neurotoxicity and severe neurotoxicity response was recorded. We implemented an NMA to identify the relative efficacy of different interventions (calcium and magnesium, glutathione, amifostine, VE, and placebo) of chemotherapy-induced chronic peripheral neurotoxicity. The endpoints of this study were overall neurotoxicity and severe neurotoxicity. The grade of neurotoxicity was classified according to several neurotoxicity assessment criteria such as the National Cancer Institution Common Toxicity Criteria (NCI-CTC) (31), World Health Organization (WHO) Criteria (32), and Oxaliplatin Special Scale (OSS) (33). Severe neurotoxicity was defined as a higher than grade 2 assessment of damage posed on the nervous system.

### Statistical Analysis

A series of standard statistical analyses were implemented during the NMA. First, if possible we conducted a direct pairwise comparison between different treatments, and used the odds ratios (ORs) of incidence and the associated 95% confidence intervals (CIs) to display the results. A lower OR indicates a better efficacy. Next, network plots were graphed to describe the scale of published studies, and the number of studies, which included a direct comparison between two specific interventions, was also labeled. After the indirect evidence between two interventions was derived from their respective comparisons with the same third party, direct and indirect evidence was combined in the network comparisons. These data were quantitatively described using the ORs of incidence and the associated 95% credible intervals (CrI). A *P*-value of less than 0.05 denotes a statistically significant difference. Furthermore, the surface under cumulative ranking curve (SUCRA) of each treatment was utilized to identify and rank the most effective drugs for both overall and severe neurotoxicity. Typically, a higher SUCRA value indicates a greater satisfaction of treatment for a certain endpoint. A cluster analysis was also performed to come up with a final recommendation. In addition, the consistency between direct and indirect evidence was assessed using node splitting plots (which compared the ORs calculated from an MA and an NMA) and heat plots (where greater color intensity suggests a higher degree of inconsistency). We also calculated the Cochran’s Q-statistics and conducted an inconsistency *I*^2^ test to evaluate heterogeneity. A *P*-value of less than 0.1 in the Cochran’s *Q*-test or greater than 50% value in the *I*^2^ value illustrated significant heterogeneity. If any significant heterogeneous evidence was noted, the random-effects model was implemented instead of the commonly used fixed-effects model. Publication bias was assessed using funnel plots. The NMA was based on Bayesian framework, and data were analyzed using the WinBUGS, R 3.2.3 (with some specific packages such as “meta,” “gemtc,” “igraph,” and “netmeta”), and STATA 13.0 software.

## Results

### Study Characteristics

**Figure 1:**
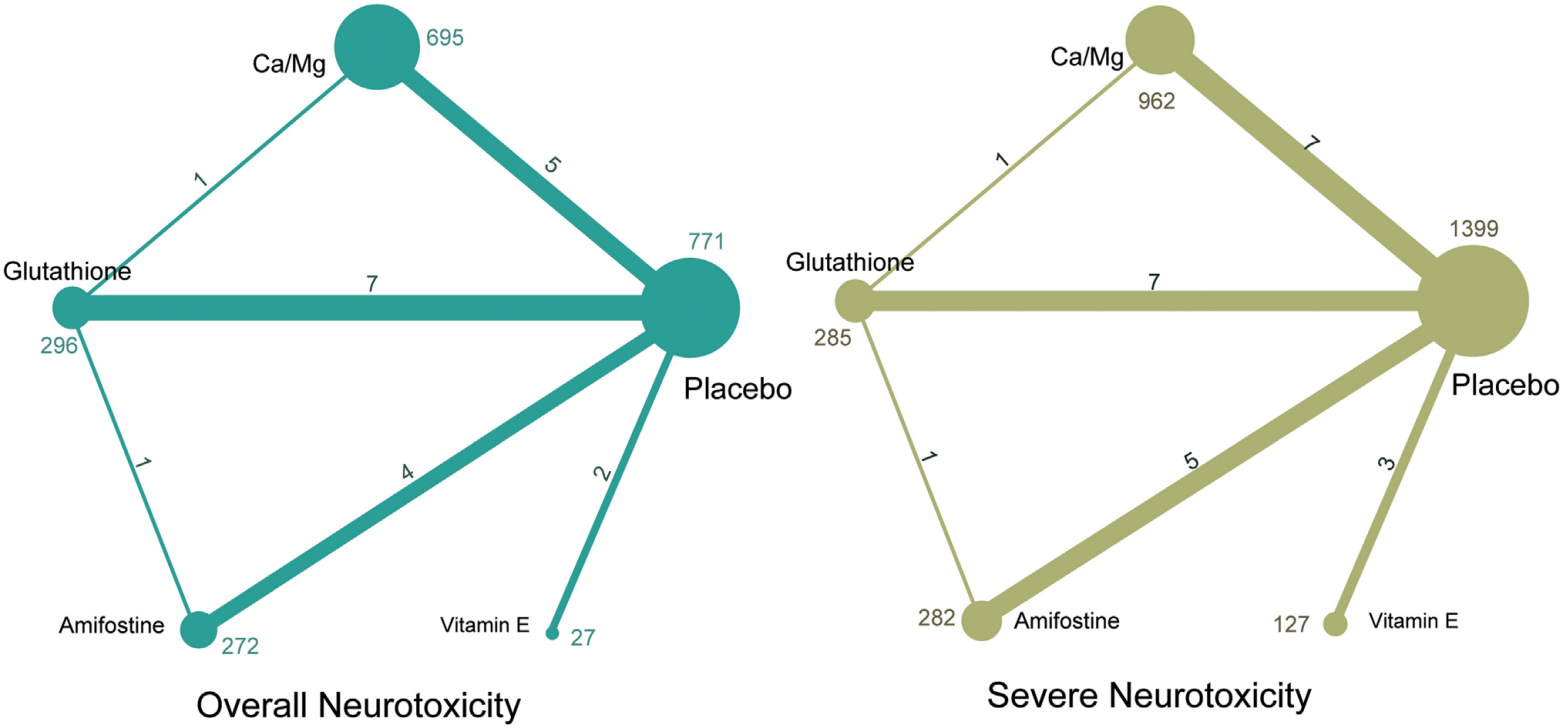
**Network of randomized controlled trials comparing different interventions of neurotoxicity**. Numbers above lines represent direct comparisons between two interventions. Numbers above dots shows the total size of interventions.

A total of 1,839 potentially relevant publications were retrieved from the Medline, Embase, and CNKI databases. A total of 348 duplicates were removed, and the remaining 1,491 records were further screened according to their titles, abstracts, and contents. Finally, 23 articles were selected to be included in the study and their publication dates ranged from 1995 to 2014 (2, 8–18, 34–44). A flowchart of identification, screening, and inclusion is shown in Figure S1 in Supplementary Material. The Jadad scale of the 23 included studies is listed in the Table S1 in Supplementary Material. This NMA involved five different treatments, including a placebo, calcium and magnesium infusion, glutathione, amifostine, and VE. The efficacy outcomes we studied were based on the ability of the above drugs to decrease overall and severe neurotoxicity in chemotherapy-treated cancer patients. The network plot in Figure 1 shows the current research situation of these treatments. After adding up the sample sizes of the same treatment group in different involved studies, the total sample size of glutathione was the largest and VE was the smallest (with only 29). Furthermore, glutathione was most frequently compared (compared with a placebo in seven studies, with Ca/Mg in one study, and with amifostine in one study). Table 1 provides basic information on the included studies.

**Table 1:** **Main characteristics of included studies**.

Study	Randomization	Blind	Standard	Patients		Group 1		Group 2		Dose	Overall neurotoxicity		Severe neurotoxicity^b^	
				Tumor site	Chemotherapy	Treatment	Size	Treatment	Size		Group 1	Group 2	Group 1	Group 2
Grothey et al. (11)	Yes	Double-blind	NCI-CTC	Colorectal	Oxaliplatin	Ca/Mg	50	Placebo	52	Ca: 1 g, Mg: 1 g	–	–	11 (22%)	21 (40%)
Dong et al. (35)	Yes	Double-blind	NCI-CTC	Gastrointestinal tract	Oxaliplatin	Ca/Mg	29	Placebo	31	Ca: 1 g, Mg: 1 g	22 (76%)	27 (87%)	4 (14%)	11 (35%)
				Gastrointestinal tract	Oxaliplatin	Glu	33	Placebo	31	Glu: 1,500 mg/m^2^	27 (82%)	27 (87%)	8 (24%)	11 (35%)
Ishibashi et al. (37)	Yes	Double-blind	NCI-CTC	Colorectal	Oxaliplatin	Ca/Mg	17	Placebo	16	Ca: 0.85 g, Mg: 0.72 g	17 (100%)	15 (94%)	1 (6%)	1 (6%)
Chay et al. (2)	Yes	Double-blind	NCI-CTC	Colorectal	Oxaliplatin	Ca/Mg	13	Placebo	14	Ca: 1 g, Mg: 1 g	7 (54%)	10 (71%)	7 (54%)	7 (50%)
Knijn et al. (9)	No	NS	NCI-CTC	Colorectal	Oxaliplatin	Ca/Mg	551	No	181	Ca: 2.25 mmol, Mg: 4 mmol, 100 ml	466 (85%)	166 (92%)	218 (40%)	81 (45%)
Loprinzi et al. (8)	Yes	Double-blind	NCI-CTC	Colon	Oxaliplatin	Ca/Mg	237	Placebo	116	Ca: 2 or 1 g/day, Mg: 2 or 1 g/day	–	–	63 (27%)	31 (27%)
Gamelin et al. (10)	No	NS	NCI-CTC	Colorectal	Oxaliplatin	Ca/Mg	96	No	65	Ca: 1 g/day, Mg: 1 g/day	19 (20%)	29 (45%)	20 (21%)	29 (45%)
Pace et al. (43)	Yes	Double-blind	NS	Lung, glioma	Cisplatin	VE	17	Placebo	24	VE: 400 mg/day	–	–	1 (6%)	10 (42%)
Argyriou et al. (18)	Yes	Single-blind	WHO	Various	Cisplatin	VE	16	No	19	VE: 600 mg/day	3 (19%)	11 (58%)	8 (50%)	7 (37%)
Kottschade et al. (39)	Yes	NS	NCI-CTC	Various	Various^a^	VE	103	Placebo	104	VE: 300 mg	–	–	33 (32%)	27 (26%)
Pace et al. (17)	Yes	NS	WHO	Various	Cisplatin	VE	13	No	14	VE: 300 mg/day	4 (31%)	12 (86%)	–	–
Cascinu et al. (12)	Yes	Double-blind	WHO	Ovarian	Cisplatin	Glu	25	Placebo	25	Glu: 1.5 g/m^2^	0 (0%)	16 (64%)	1 (4%)	13 (52%)
Cascinu et al. (13)	Yes	Double-blind	NCI-CTC	Colorectal	Oxaliplatin	Glu	26	Placebo	26	Glu: 1.5 g/m^2^	9 (35%)	15 (58%)	3 (12%)	8 (31%)
Milla et al. (42)	Yes	NS	NCI-CTC	Colorectal	Oxaliplatin	Glu	14	Placebo	13	Glu: 1,500 mg/m^2^	14 (100%)	13 (100%)	7 (50%)	13 (100%)
Liu et al. (41)	Yes	NS	OSS	Colorectal	Oxaliplatin	Glu	54	Placebo	51	Glu: 1.9 g/day	37 (69%)	46 (90%)	10 (19%)	16 (31%)
Li (40)	Yes	NS	OSS	NS	Oxaliplatin	Glu	40	Placebo	40	Glu: 1,200 mg/d	3 (8%)	21 (53%)	2 (5%)	11 (28%)
Smyth et al. (44)	Yes	No	NCI-CTC	Ovarian	Cisplatin	Glu	74	Placebo	77	Glu: 3 g/m^2^	29 (39%)	36 (47%)	2 (3%)	2 (3%)
Gallardo et al. (36)	Yes	Single-blind	NCI-CTC	Cervical	Cisplatin	Ami	10	No	10	Ami: 825 mg/m^2^	–	–	1 (10%)	4 (40%)
Kanat et al. (38)	Yes	NS	NCI-CTC	Lung	Carboplatin and paclitaxel	Ami	19	No	19	Ami: 910 mg/m^2^	8 (42%)	18 (95%)	2 (11%)	18 (95%)
Kemp et al. (14)	Yes	NS	NCI-CTC	Ovarian	Cisplatin	Ami	122	No	120	Ami: 910 mg/m^2^	67 (55%)	81 (68%)	38 (31%)	50 (42%)
Lu et al. (15)	Yes	NS	NCI-CTC	Colorectal, Gastric	Oxaliplatin	Ami	46	Glu	46	Ami: 500 mg/m^2^/4 week, Glu: 1,500 mg/m^2^	6 (14%)	37 (86%)	1 (2%)	8 (19%)
De Vos et al. (16)	Yes	NS	NCI-CTC	Ovarian	Carboplatin	Ami	45	No	45	Ami: 740 mg/m^2^	38 (84%)	42 (93%)	7 (16%)	17 (38%)
Chen et al. (34)	Yes	NS	OSS	Colorectal, Gastric	Oxaliplatin	Ami	40	Placebo	40	Ami: 500 mg/m^2^/treatment	12 (30%)	26 (65%)	7 (18%)	14 (35%)

*NCI-CTC, National Cancer Institution Common Toxicity Criteria; WHO, World Health Organization; OSS, Oxaliplatin Special Scale; NS, not specified; VE, vitamin E; Glu, glutathione; Ami, amifostine*.

*^a^Taxanes, cisplatin, carboplatin, oxaliplatin, or combination*.

*^b^Grade ≥ 2*.

### Overall Neurotoxicity

**Figure 2:**
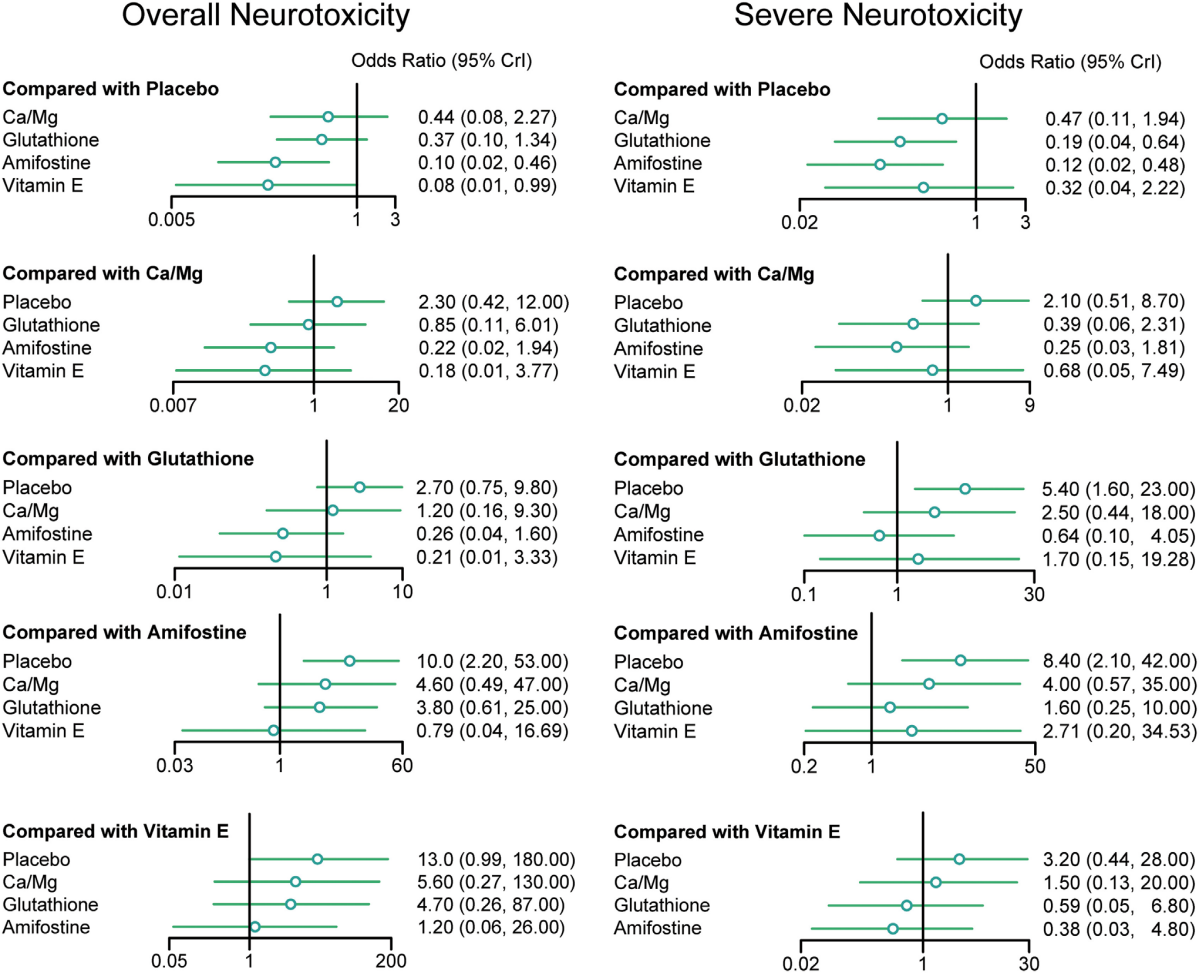
**Odds ratios (95% credential intervals) for network comparison of neurotoxicity treatments**.

According to the direct comparison results between different neurotoxicity treatments (Table 2), subjects treated with glutathione, VE, and amifostine showed a reduced risk of overall neurotoxicity compared with those treated with the placebo (glutathione: OR = 0.64, 95% CI: 0.47–0.86; VE: OR = 0.34, 95% CI: 0.12–0.91 and amifostine: OR = 0.73, 95% CI: 0.54–0.99). Furthermore, amifostine appears to be superior to glutathione in decreasing the risk of overall neurotoxicity (OR = 0.14, 95% CI = 0.05–0.36). As an NMA combines both direct and indirect evidence, the results are similar but not exactly the same (Table 3 and Figure 2). Patients treated with amifostine or VE exhibited a significantly lower risk of overall neurotoxicity than those treated with the placebo (amifostine: OR = 0.10, 95% CrI: 0.02–0.46; VE: OR = 0.08, 95% CrI: 0.01–0.99).

**Table 2:** **Direct pairwise comparison results of neurotoxicity treatments**.

Comparison	Overall neurotoxicity	Severe neurotoxicity^a^
Ca/Mg vs. placebo	0.86 (0.70, 1.06)	1.55 (1.27, 1.91)
Glutathione vs. placebo	**0.64 (0.47, 0.86)**	**0.42 (0.27, 0.67)**
Glutathione vs. Ca/Mg	1.01 (0.45, 2.26)	1.63 (0.43, 6.14)
Vitamin E vs. placebo	**0.34 (0.12, 0.91)**	0.84 (0.51, 1.40)
Amifostine vs. placebo	**0.73 (0.54, 0.99)**	**0.53 (0.37, 0.78)**
Amifostine vs. glutathione	**0.14 (0.05, 0.36)**	**0.11 (0.01, 0.91)**

*Bold font indicates statistically significant difference*.

*The data are odds ratio and 95% confidence interval*.

*^a^Grade ≥ 2*.

**Table 3:** **The surface under cumulative ranking curve results of neurotoxicity treatments**.

Treatment	Overall neurotoxicity	Severe neurotoxicity^a^
Placebo	0.060	0.062
Ca/Mg	0.375	0.365
Glutathione	0.425	0.710
Amifostine	0.822	0.855
Vitamin E	0.822	0.507

*^a^Grade ≥ 2*.

### Severe Neurotoxicity

The direct comparison results with respect to the risk of severe neurotoxicity are displayed in Table 2. Patients treated with glutathione or amifostine exhibited a significantly reduced risk of severe neurotoxicity compared with those treated with the placebo (glutathione: OR = 0.42, 95% CI: 0.27–0.67; amifostine: OR = 0.53, 95% CI: 0.37–0.78). Moreover, patients treated with amifostine appear to have a lower risk of severe neurotoxicity than those treated with glutathione (OR = 0.11, 95% CI: 0.01–0.91). The corresponding NMA results are presented in Table 4 and Figure 2: glutathione and amifostine present notable superiority compared to the placebo in preventing severe neurotoxicity (glutathione: OR = 0.19, 95% CrI: 0.04–0.64; amifostine: OR = 0.12, 95% CrI: 0.02–0.48).

**Table 4:** **Network meta-analysis results of neurotoxicity treatments**.

	Overall neurotoxicity				
Severe neurotoxicity^a^	**Placebo**	0.44 (0.08, 2.27)	0.37 (0.10, 1.34)	**0.10 (0.02, 0.46)**	**0.08 (0.01, 0.99)**
	0.47 (0.11, 1.94)	**Ca/Mg**	0.85 (0.11, 6.01)	0.22 (0.02, 1.94)	0.18 (0.01, 3.77)
	**0.19 (0.04, 0.64)**	0.39 (0.06, 2.31)	**Glutathione**	0.26 (0.04, 1.60)	0.21 (0.01, 3.33)
	**0.12 (0.02, 0.48)**	0.25 (0.03, 1.81)	0.64 (0.10, 4.05)	**Amifostine**	0.79 (0.04, 16.69)
	0.32 (0.04, 2.22)	0.68 (0.05, 7.49)	1.70 (0.15, 19.28)	2.71 (0.20, 34.53)	**Vitamin E**

*Bold font indicates statistically significant difference and nothing special about the gray shade*.

*^a^Grade ≥ 2*.

### Consistency and Conformity Assessment

**Figure 3:**
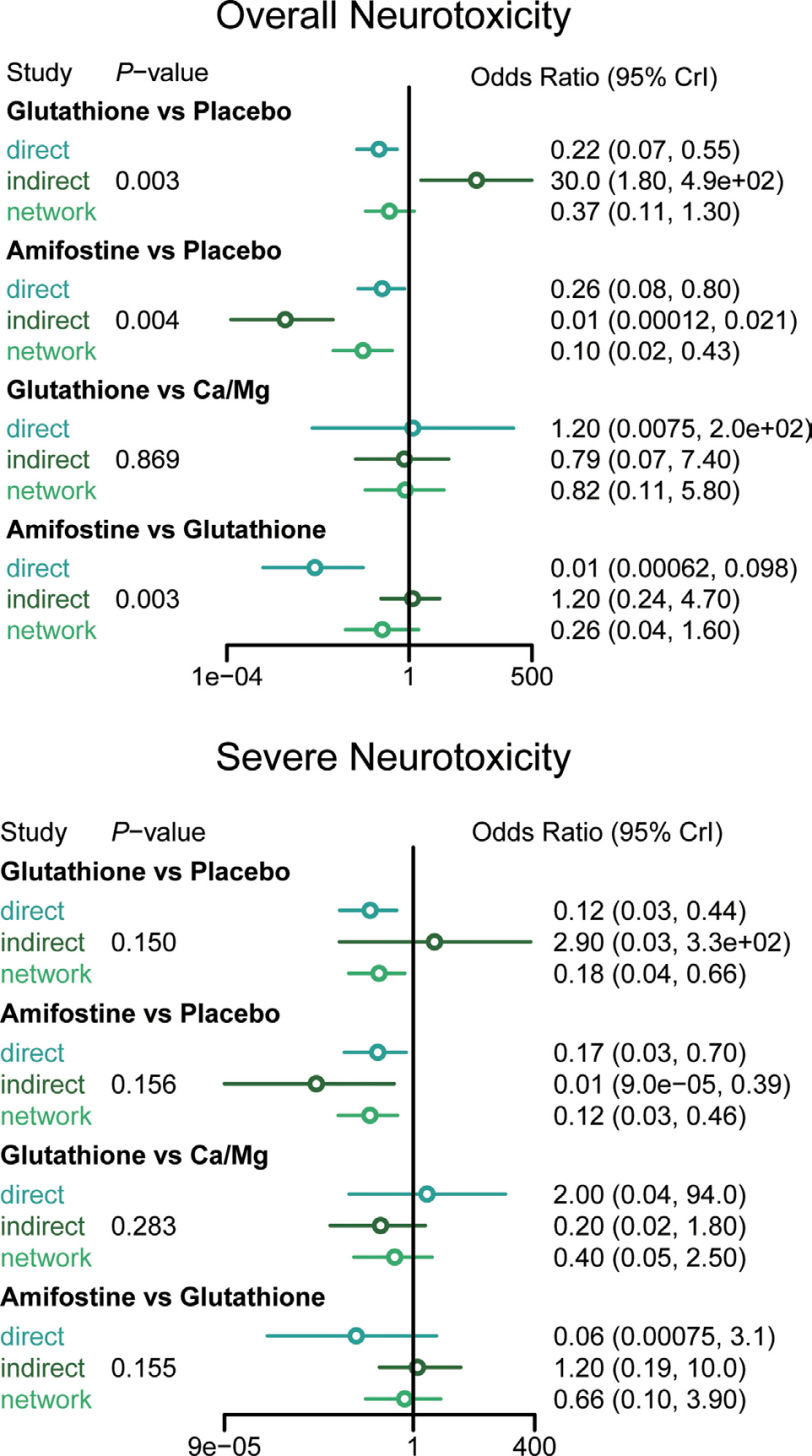
**Node splitting results according to type of interventions for all clinical outcomes**.

**Figure 4:**
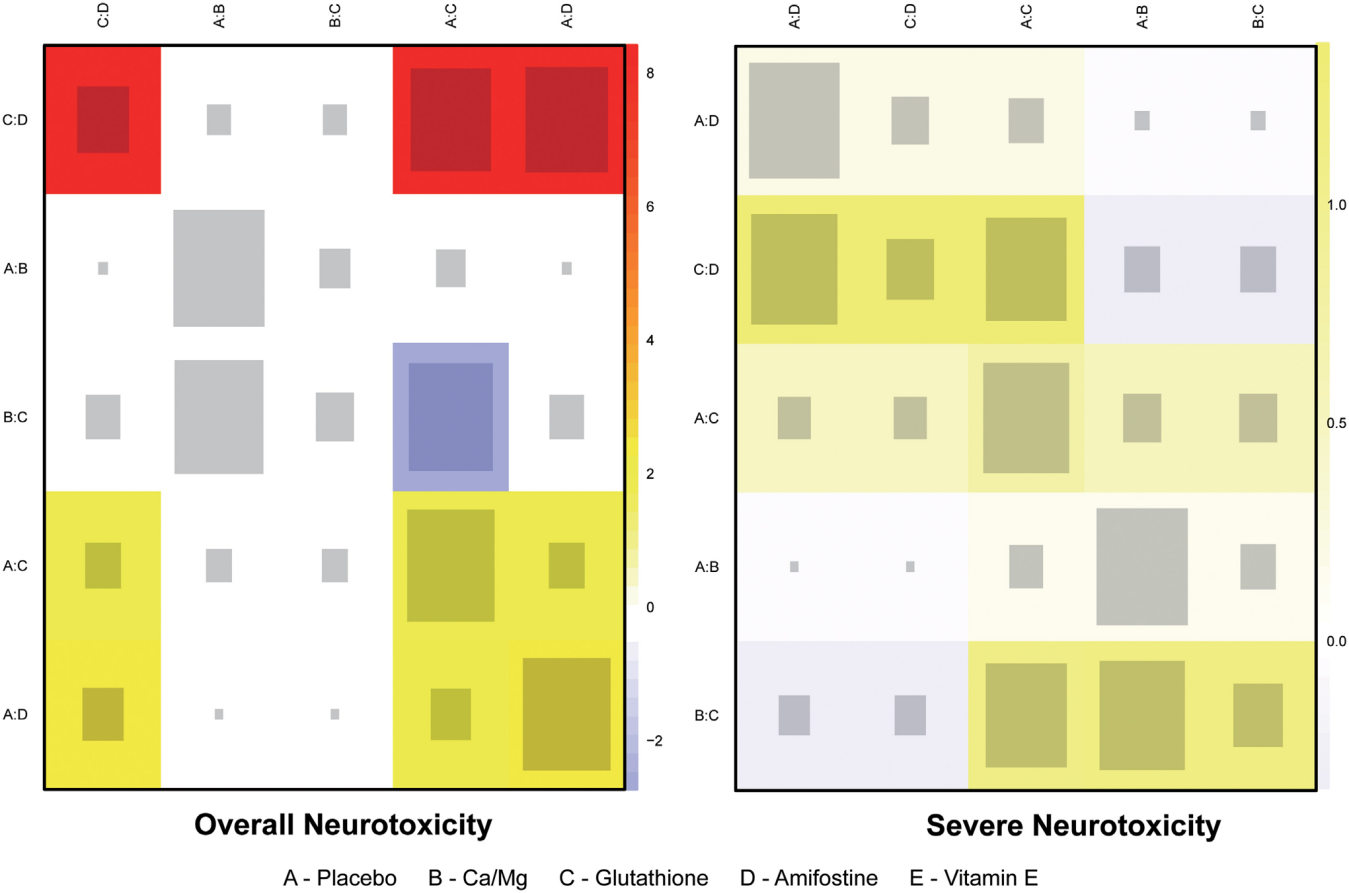
**Heat plot for neurotoxicity treatments**. The area of the gray squares displays the contribution of the direct estimate in design d (shown in the column) to the network estimate in design (shown in the row). The colors are associated with the change in inconsistency between direct and indirect evidence (shown in the row) after detaching the effect (shown in the column). Blue colors indicate an increase, and warm colors indicate a decrease (the stronger the intensity of the color, the stronger the change).

The node splitting method was used to assess the consistency of direct and indirect evidence (Figure 3). A *P*-value of <0.05 indicates a significant inconsistency between direct and indirect evidence. A significant inconsistency with respect to the risk of overall neurotoxicity was detected in the comparison between glutathione and placebo, amifostine and placebo, and amifostine and glutathione. No significant inconsistency was observed in the comparisons with respect to the risk of severe neurotoxicity. We also produced heat plots in order to assess the conformity of direct and indirect evidence (Figure 4). A deeper color indicates a higher inconsistency. A significant inconsistency in overall neurotoxicity was found in the comparisons of glutathione and placebo, amifostine and placebo, and amifostine and glutathione. No significant inconsistency was observed in the comparisons for severe neurotoxicity.

### Ranking and Cluster Analysis

**Figure 5:**
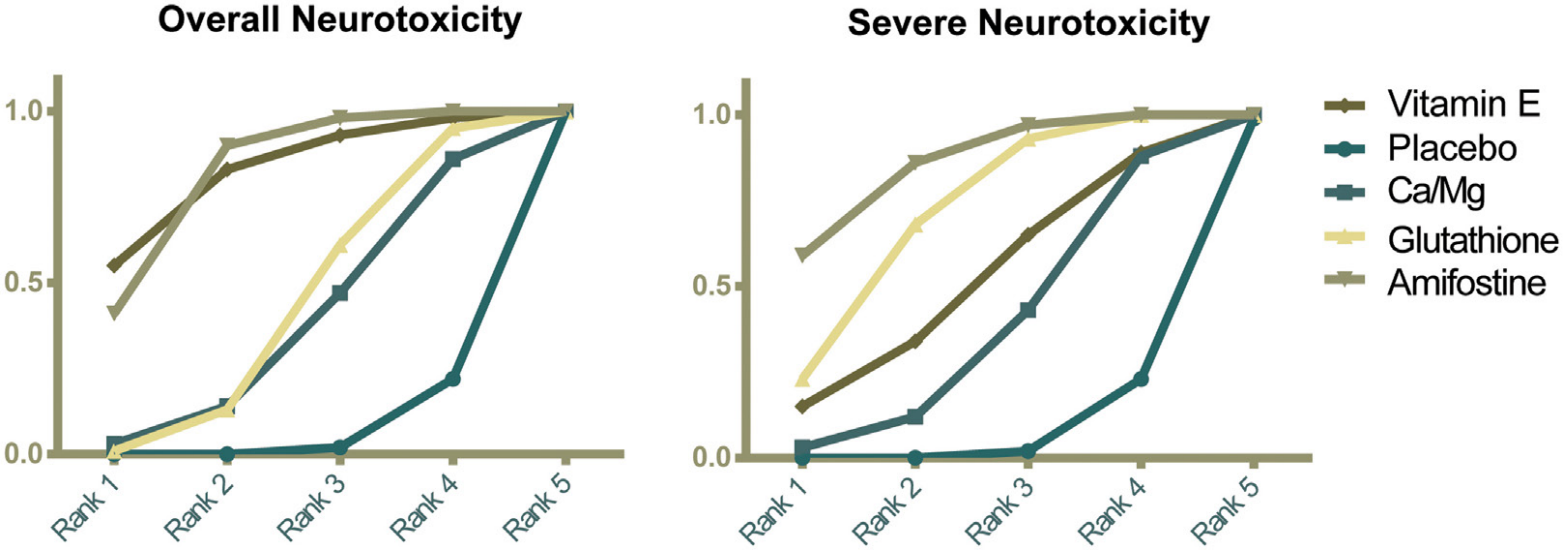
**Ranking graphs showing probability of each strategy having each specific rank (1–5) for outcomes**. Ranking indicates the probability to be the best treatment, the second best, and so on. Rank 1 is best and Rank 5 is worst.

**Figure 6:**
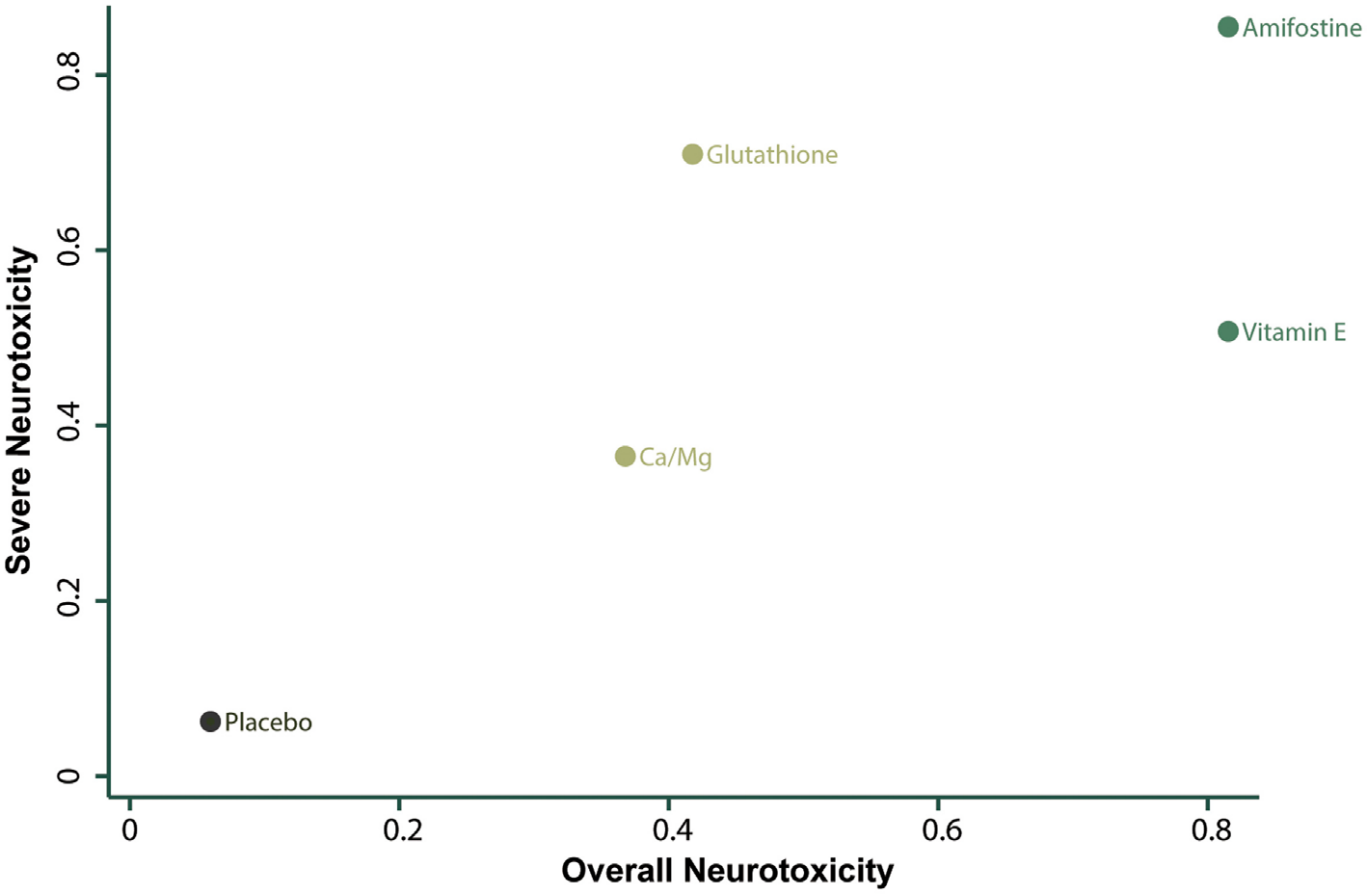
**Cluster analysis**.

The corresponding rank of each intervention was determined basing on their SUCRA value (Table 4 and Figure 5). A higher SUCRA indicates a greater efficacy. Amifostine and VE proved to be the 2 most preferable treatments with respect to overall neurotoxicity (SUCRA = 0.82 for both), while amifostine with the highest SUCRA of 0.86 was the top choice for severe neurotoxicity. We also attempted to categorize the five interventions (placebo, Ca/Mg, glutathione, VE, and amifostine) using a cluster analysis (Figure 6). In the cluster analysis plot, treatments were set as points according to their SUCRA values regarding axis events. Amifostine scored best in terms of both overall and severe neurotoxicity.

### Publication Bias

We conducted an assessment of publication bias using funnel plots (Figure S2 in Supplementary Material). According to the symmetrical distribution of the points (where each point denotes a corresponding involved study), no evidence of publication bias was observed for both overall and severe neurotoxicity.

## Discussion

### Primary Findings

A systematic analysis was performed in order to determine the best therapeutic intervention for CICNT in cancer patients. The efficacy of different treatments was evaluated based on their ability to prevent overall and severe neurotoxicity. The overall neurotoxicity network comparison results demonstrated that amifostine and VE both have significantly lower OR values compared to the placebo, indicating a high efficacy. In regard to preventing severe neurotoxicity, glutathione and amifostine exhibited superiority. The SUCRA and cluster plot outcomes of the five interventions (including the placebo as a control) also provided us with useful information. They demonstrated that amifostine and VE were the two top recommended interventions for overall neurotoxicity and amifostine and glutathione were favored for severe neurotoxicity. Thus, we could confidently conclude that amifostine and VE should be recommended in treating overall neuropathy and that amifostine and glutathione should be recommended to treat severe neurotoxicity. Amifostine is our top recommendation for treating neurotoxicity. The excellent performance of amifostine in treating CICNT may come from its ability to eliminate the harmful oxidants derived from the interaction of oxygen radicals and neurotoxicity-related DNA groups. It does this by competing with oxygen or oxygen radicals for the binding to DNA groups. This mechanism is similar to the function of endogenous thiol (45). Furthermore, due to the intracellular micro-environmental difference between normal and tumor cells, amifostine can be absorbed into normal cells (46) and have a protective effect.

### Direct and Indirect Evidence in this Study

An NMA synthesizes non-conclusive evidence from clinical trials to construct a network which compares multiple treatments simultaneously. This network is mainly based on direct evidence; indirect evidence is obtained by forming opinions on related direct evidence. Each treatment involved in the NMA would be compared using both direct and indirect evidence or just indirect evidence. However, a limitation of NMA is that there can be significant incoherence between direct and indirect comparisons (referred to as inconsistency) (47). The results of the node splitting and heat plots revealed that the direct comparison results of amifostine and glutathione against the placebo significantly deviated from the corresponding indirect comparison results. The comparison between amifostine and glutathione also displayed a striking inconsistency between the two types of evidence. This may be due to the limited direct comparison evidence we were able to obtain on amifostine and glutathione (only 1 included study with only 46 patients in each group) (15). However, the direct and indirect evidence of other intervention comparisons were consistent and validate the consistency model applied in this NMA.

### Consistencies and Discrepancies with Other Studies

Our result that amifostine is extremely effective in treating both overall and severe neurotoxicity is supported by the findings of various clinical trials (14, 16, 36). However, there is one exception that reports that amifostine is not able to have a preventive role in neuropathy (38). This inconsistency may result from the relatively small sample size (19) and special type of chemotherapy (carboplatin and paclitaxel) used in the study. Most previous studies reported that the neuroprotective ability of VE can protect cancer patients from experiencing chemotherapy-induced neurotoxicity effectively and safely (17, 18, 43). In this study, VE also showed superiority in preventing overall neurotoxicity (though with a considerably large 95% CrI). Glutathione is another intervention suggested to be a protective intervention for chemotherapy-induced neurotoxicity. We found that glutathione had above average efficacy in reducing severe neurotoxicity. This result is also emphasized in other studies (12, 13). The inner mechanism of its ability to weaken neurotoxicity is believed to be a function of the thionucleophilic region located inside the glutathione. Its high heavy metal-binding ability enables it to prevent the accumulation of platinum (7). Calcium and magnesium infusion (Ca/Mg) is the most popular regimen used to prevent and treat neurotoxicity. However, its real efficacy still remains disputed. Individual trials conducted over the last few years imply that it is an effective intervention for dealing with oxaliplatin-induced cumulative neurotoxicity in colon cancer (11) and that it can also reduce the probability of all-grade neurotoxicity (9). Furthermore, the efficacy of Ca/Mg has been verified by a number of MA studies. For example, one such study concluded that subjects treated with Ca/Mg displayed a significantly decreased risk of developing both grade 1 and grade 2 oxaliplatin-induced neurotoxicity (21). Another MA study had a similar conclusion (22), reporting that Ca/Mg tends to lower the incidence of oxaliplatin-induced acute and cumulative neurotoxicity without remarkably altering the validity of chemotherapy. On the other hand, some studies (2, 8) indicated that Ca/Mg had no significantly higher efficacy compared to the placebo. In fact, as no remarkable difference was detected between the Ca/Mg and placebo groups, this study also did not support the efficacy of Ca/Mg. The application of Ca/Mg may be not as ideal and further study is required.

### Advantages and Limitations

No previous MA study has recommended the ideal treatment for CICNT. However, through the application of an NMA, this research makes a systematic simultaneous comparison of multiple treatments and makes a recommendation on the ideal treatment. Both direct and indirect evidence contributed to comprehensively assessing the effectiveness of each drug. There is often a lack of direct evidence between active interventions and this limits further drug evaluation. However, this can be remedied by an NMA by utilizing indirect comparisons. Most of the research involved in this study made comparisons between a certain drug and the placebo (only one compared amifostine and glutathione directly). However, by constructing a network that connects all interventions with the supplement of indirect evidence, we were able to compare the relative efficacy between any two interventions.

Nevertheless, there are still some limitations of this study. First, this NMA only included 23 studies and this relatively small study size had a considerable width of 95% CrI in some drugs (VE). Therefore, there was a large variance in the amount of data related to different interventions. The recent clinical trials conducted on CICNT are also very limited. Ca/Mg has long been used as a common CICNT treatment. If our NMA included more studies, the results could be more representative and powerful. Second, during the process of literature review and data extraction, we noted that there were various standards of neurotoxicity assessment such as the NCI-CTC (31), WHO Criteria (32), and the OSS (33). This inconsistency in evaluating criteria for neurotoxicity, along with the differences in patient background such as tumor types, stage, and type of chemotherapy received can skew results.

### Prospect

Much research regarding the ideal treatment for CICNT is required. In this study, we defined a neurotoxicity of above grade 0 as overall neurotoxicity and above grade 2 as severe neurotoxicity. However, in a practical clinical situation, the average danger of overall and severe neurotoxicity is much different. This suggests that different corresponding weights should be considered in NMAs to give a more realistic recommendation (48). In addition, as a result of insufficient data in the included studies, we were unable to conduct an analysis of the safety characteristics of each drug. Further studies concerning the safety of each treatment is required.

## Conclusion

Current clinical studies and MAs have not yet determined one effective and practical intervention for CICNT. Our NMA aimed to reveal the ideal regimen of CICNT treatment. Amifostine is our top recommendation as it has the highest efficacy for both overall and severe neurotoxicities. The second choice for treating severe neurotoxicity is glutathione and VE for overall neurotoxicity. As we were limited by data, we did not compare the safety outcome of each therapy; therefore, there is still an urgent need for future research.

## Author Contributions

Conception and design: WY. Acquisition of data and analysis and interpretation of data: XF, HW, and JL. Drafting the article: XF, CW, LM, and QM. Critically revising the article: CW, LM, and QM. Administrative/technical/material support: WY.

## Conflict of Interest Statement

The authors declare that the research was conducted in the absence of any commercial or financial relationships that could be construed as a potential conflict of interest. The reviewer, DM, and handling editor declared their shared affiliation and the handling editor states that the process nevertheless met the standards of a fair and objective review.

## Supplementary Material

Figure S1 **Literature flowchart**.

Figure S2 **Publication bias**.

Table S1 **Jadad scale of 23 included studies**.
