# Supplementary figures and images for: Efficacy of Drug Interventions for Chemotherapy-Induced Chronic Peripheral Neurotoxicity: A Network Meta-analysis
Source: Front Neurol. 2017 Jun 8;8:223. doi: 10.3389/fneur.2017.00223 (PMC5462987; doi:10.3389/fneur.2017.00223)

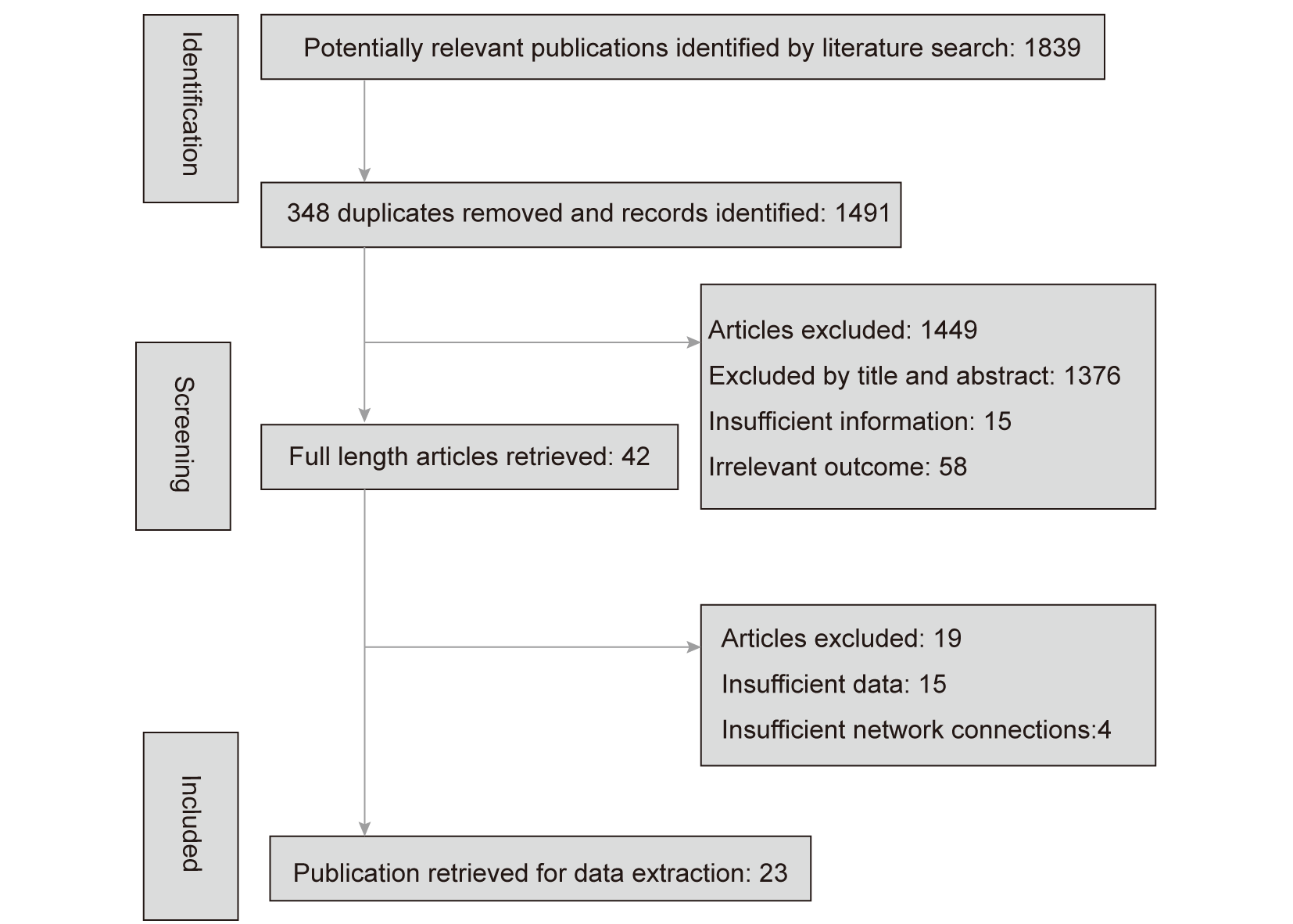

Supplement: Figure S1 — Literature flowchart. [file Image_1.TIF]

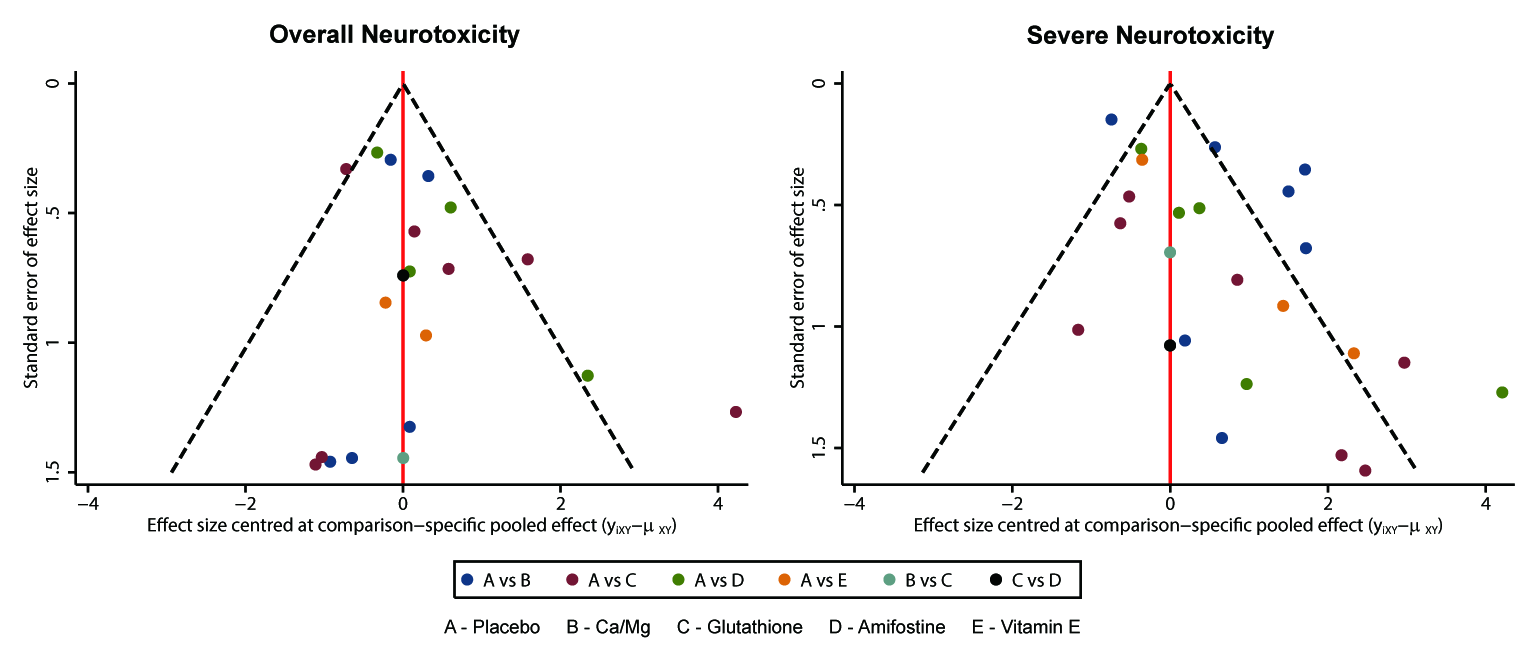

Supplement: Figure S2 — Publication bias. [file Image_2.TIF]
