# Supplementary material for: Efficacy of Drug Interventions for Chemotherapy-Induced Chronic Peripheral Neurotoxicity: A Network Meta-analysis
Source: Front Neurol. 2017 Jun 8;8:223. doi: 10.3389/fneur.2017.00223 (PMC5462987; doi:10.3389/fneur.2017.00223)
Supplement: Table S1 — Jadad scale of 23 included studies. [file Table_1.DOCX]

**Table S1. Jadad Scale of 23 included study**

| **Study or Subgroup** | **Design** | **Blinding** | **Withdrawal** |
| --- | --- | --- | --- |
| Grothey, 2011 | 1 | 2 | 1 |
| Dong, 2010 | 1 | 2 | 1 |
| Ishibashi, 2010 | 2 | 2 | 1 |
| Chay, 2010 | 1 | 2 | 1 |
| Knijn, 2011 | 0 | 0 | 1 |
| Loprinzi, 2014 | 2 | 2 | 1 |
| Gamelin, 2004 | 0 | 0 | 1 |
| Pace, 2010 | 2 | 2 | 1 |
| Argyriou, 2006 | 2 | 1 | 1 |
| Kottschade, 2011 | 2 | 0 | 1 |
| Pace, 2003 | 1 | 0 | 1 |
| Cascinu, 1995 | 2 | 2 | 1 |
| Cascinu, 2002 | 2 | 2 | 1 |
| Milla, 2009 | 1 | 0 | 1 |
| Liu, 2011 | 2 | 0 | 1 |
| Li, 2014 | 2 | 0 | 1 |
| Smyth, 1997 | 2 | 0 | 1 |
| Gallardo, 1999 | 2 | 1 | 1 |
| Kanat, 2003 | 1 | 0 | 1 |
| Kemp, 1996 | 1 | 0 | 1 |
| Lu, 2008 | 2 | 0 | 1 |
| De Vos, 2005 | 1 | 0 | 1 |
| Chen, 2011 | 2 | 0 | 1 |
